# The prognostic significance of duodenal wall invasion in pancreatic adenocarcinoma

**DOI:** 10.1186/s12957-023-02962-6

**Published:** 2023-03-06

**Authors:** Ahmad Alkhasawneh, Tasnuva Rashid, Ibraheem Mohammed, Basma Elhaddad, Hassan Al-Balas, Mayur Virarkar, Ziad Awad, Brett Baskovich, Arun Gopinath

**Affiliations:** 1 UF Health Jacksonville, 655 W 8th st, Jacksonville, FL USA; 2 Jordan University of Science and Technology, Irbid, Jordan; 3 University of Florida College of Medicine, Jacksonville, 653 West 8th Street, Jacksonville, FL 32209 USA; 4 Mount Saini Health System, New York, NY USA

**Keywords:** Pancreas, Duodenum, Adenocarcinoma, Invasion, Whipple, Pancreatectomy

## Abstract

**Objective:**

The most recent edition of the American Joint Committee on Cancer Staging Manual (AJCC, 8th edition) relies only on tumor size for staging resectable pancreatic adenocarcinoma, and the presence of duodenal wall invasion (DWI) no longer has an impact on staging. However, very few studies have evaluated its significance. In this study, we aim to evaluate the prognostic significance of DWI in pancreatic adenocarcinoma.

**Methods:**

We reviewed 97 consecutive internal cases of resected pancreatic head ductal adenocarcinoma, and clinicopathologic parameters were recorded. All cases were staged according to the 8th edition of AJCC, and the patients were divided into two groups based on the presence or absence of DWI.

**Results:**

Out of our 97 cases, 53 patients had DWI (55%). In univariate analysis, DWI was significantly associated with lymphovascular invasion and lymph node metastasis (AJCC 8th edition pN stage). In univariate analysis of overall survival, age > 60, absence of DWI, and African American race were associated with worse overall survival. In multivariate analysis, age > 60, absence of DWI, and African American race were associated with worse progression-free survival and overall survival.

**Conclusion:**

Although DWI is associated with lymph node metastasis, it is not associated with inferior disease-free/overall survival.

## Introduction

Pancreatic adenocarcinoma is one of the leading causes of cancer-related death [1, 2], and adverse prognostic factors in pancreatic carcinoma include perineural invasion, lymphovascular invasion, advanced stage, and lymph node metastasis and the number of involved lymph nodes [3–6].

Staging is an important factor for determining clinical management and predicting outcomes. In the previous American Joint Committee on Cancer (AJCC) staging system (7th edition), a cutoff of 2 cm was used to separate tumors into pT1 and pT2 when the tumor is confined to the pancreas. Regardless of the size or the presence of invasion beyond the pancreas (i.e., extrapancreatic tumor extension into the duodenum, peripancreatic tissue, or adjacent organs), a tumor was staged as pT3 in the absence of celiac axis or superior mesenteric artery involvement by tumor (which is staged as pT4). In the most recent AJCC staging criteria (8th edition), a tumor is staged as pT3 solely based on the size of the tumor being above 4 cm. Histologic parameters like duodenal wall invasion (DWI) and extrapancreatic extension were removed from the pathologic tumor staging.

There is a paucity of studies evaluating the clinical significance of duodenal invasion in pancreatic adenocarcinoma, and there is conflicting data about its prognostic significance [7–9]. In this study, we aim to evaluate the clinicopathologic characteristics of pancreatic adenocarcinoma and the prognostic significance of duodenal wall invasion.

## Methods

This study was approved by the Institutional Review Board at our institution (IRB #202,100,888). Pancreatic adenocarcinoma patients with different TNM stages who had undergone Whipple resection and/or distal pancreatectomy at a tertiary care hospital during the period between January 2008 and May 2021 were reviewed in the study, and patients who underwent Whipple resection with or without total pancreatectomy who survived more than 30 days following surgery were analyzed. The following information was obtained from the electronic medical record and tumor registry at our institution: age, gender, and clinical follow-up concerning progression, recurrence, and survival (as of July 1, 2021).

All gross descriptions and hematoxylin and eosin slides were reviewed by three board-certified anatomic pathologists with experience in gastrointestinal pathology (AA, BB, AG) for the following parameters: tumor size (maximum tumor dimension in the pathology report), histologic grade, margin status (R0: negative and R1: positive or less 1 mm for the retroperitoneal margin), the presence of intraductal papillary mucinous neoplasm (IPMN), perineural invasion (PNI), lymphovascular invasion (LVI), DWI (involvement of muscularis propria of the duodenal wall and/or ampullary involvement), extrapancreatic common bile duct invasion by tumor, the number of examined lymph nodes, and the number of involved lymph nodes. All cases were grossed according to our institution’s protocol which includes at least 1 routine section from ampulla (including duodenal wall and pancreas), and cases were staged according to the 8th edition of the AJCC.

The patients were divided into 2 groups: group 1 with DWI and group 2 without DWI, and the clinicopathologic features were compared between the two groups. Descriptive summaries included frequencies and percentages for categorical variables and means (and range) for continuous variables. Univariate analyses were done to compare group 1 and group 2. Means (and ranges) of continuous variables with normal distributions were compared using the two-tailed Student *t*-test. Pearson’s chi-squared test or Fisher’s exact test were used as applicable to compare the categorical variables. Multivariate logistic regression with unadjusted and adjusted models was run to identify variables significantly associated with DWI.

Overall survival (OS) was calculated from the date of surgical resection to the date of death or last follow-up. Progression-free survival (PFS) was calculated from the date of surgical resection to the date of first recurrence or death, whichever came first. The OS rate and PFS time were calculated using Kaplan–Meier curves, and the log-rank test was used to determine the statistical significance of differences. Multivariate Cox regression analysis was conducted to identify whether DWI or any other parameters in the model were significantly associated with OS and PFS post-pancreatic cancer surgery using unadjusted and adjusted models. STATA/BE 17 was used for data management and statistical analysis. A *P*-value ≤ 0.05 was considered statistically significant.

## Results

### Patient characteristics and survival

There were 102 patients who underwent Whipple resection for pancreatic adenocarcinoma at our institution in 2008–2021. Of these, 97 patients were alive 30 days following surgery and were included in the study.

The mean age of the patients was 66.2 years (range 43–89); 40% were females and 60% were males. Thirty-two patients (33%) died during the study duration with a mean survival of 21 months (range: 1–144 months) and a mean PFS of 18.6 months (range: 1–93 months).

Out of our 97 pancreatic head adenocarcinoma cases, 53 patients had DWI (55%). Analysis of factors affecting duodenal wall invasion by tumor is shown in Table 1. In univariate analysis, DWI was significantly associated with lymph node metastasis (pN stage, *P* < 0.01), lymphovascular invasion (*P* = 0.01), and near significant better survival outcome (Table 2). In multivariate analysis, however, DWI was significantly associated with lymph node involvement (*P* = 0.02) and fewer deaths (*P* = 0.03).

**Table 1:** Descriptive analysis of factors affecting duodenal wall invasion by tumor

	**Duodenal invasion present** **(*n* = 53)**	**Duodenal invasion absent** **(*n* = 44)**	***P*-value**
**Age**			
**Mean age**	67.3 (59–76)	65.2 (55–76)	0.14
**Age ≤ 60**	11 (21)	15 (34)	
**Age > 60**	42 (79)	29 (66)	
**Gender**			
**Male**	36 (68)	24 (55)	0.18
**Female**	17 (32)	20 (45)	
**Race**			
**White**	38 (83)	32 (76)	0.61
**African American**	6 (13)	6 (14)	
**Others**	2 (4)	4 (10)	
**Tumor size**^**a**^	3.49 (3.05–3.92)	3.30 (2.54–4.06)	0.65
**Lymphovascular invasion**			
**Absent**	10 (19)	17 (39)	0.03^*^
**Present**	43 (81)	27 (61)	
**Perineural invasion**			
**Absent**	5 (9)	10 (23)	0.07
**Present**	48 (91)	34 (77)	
**Extra pancreatic bile duct invasion**			
**Absent**	42 (79)	37 (84)	0.54
**Present**	11 (21)	7 (16)	
**Ampullary invasion**^**a**^			
**Absent**	23 (43)	40 (91)	0.00^*^
**Present**	30 (57)	4 (9)	
**IPMN-associated adenocarcinoma**			
**Absent**	46 (87)	36 (82)	0.50
**Present**	7 (13)	8 (18)	
**Margin status**			
**Negative**	39 (74)	29 (66)	0.41
**Positive**	14 (26)	15 (34)	
**AJCC 8th edition—tumor stage**			
**pT1**	8 (15)	12 (27)	0.30
**pT2**	30 (57)	23 (52)	
**pT3**	15 (28)	9 (21)	
**AJCC 8th edition—lymph node stage**			
**pN0**	8 (15)	18 (41)	0.01^*^
**pN1**	26 (49)	18 (41)	
**pN2**	19 (36)	8 (18)	
**Tumor grading**			
**G1**	1 (2)	4 (9)	0.19
**G2**	36 (68)	31 (70)	
**G3**	16 (30)	9 (21)	
**Lymph node involvement**			
**Absent**	8 (15)	18 (41)	0.004^*^
**Present**	45 (85)	26 (59)	
**Neoadjuvant therapy**^**a**^			
**Absent**	34 (69)	24 (67)	0.79
**Present**	15 (31)	12 (33)	
**Recurrence/metastasis**			
**Absent**	40 (75)	33 (75)	0.95
**Present**	13 (25)	11 (25)	
**Survival status**			
**Alive**	41 (77)	26 (59)	0.053
**Death**	12 (23)	18 (41)	
**Progression-free survival (in months)**	21.33 (14.78–28.17)	15.13 (9.7–20.49)	0.15
**Overall survival (in months)**	24.45 (16.39–32.50)	16.86 (10.78–22.94)	0.14

^a^Missing values—neoadjuvant treatment (12)

^*^Significant values (*p* ≤ 0.05)

*%* column percentage

**Table 2:** Unadjusted and multivariate adjusted model for association of duodenal invasion with adverse features of pancreatic cancer

Adverse features of pancreatic cancer	**Duodenal invasion, OR (*P*-value)**	
	**Univariate model**	**Multivariate model**^**a**^
Age	**1.97 (*P* = 0.14)**	**3.53 (*P* = 0.07)**
Gender	**1.49 (*P* = 0.33)**	**1.81 (*P* = 0.34)**
Race	**0.70 (*P* = 0.61)**	**1.09 (*P* = 0.88)**
Lymphovascular invasion	2.71 (*P* = 0.03)^*^	**0.83 (*P* = 0.84)**
Lymph node involvement	3.89 (*P* = 0.004)^*^	9.17 (*P* = 0.02)^*^
Perineural involvement	**2.82 (*P* = 0.07)**	**1.04 (*P* = 0.96)**
CBD involvement	**1.38 (*P* = 0.54)**	**0.34 (*P* = 0.16)**
IPMN-associated adenocarcinoma	**0.68 (*P* = 0.50)**	**0.18 (*P* = 0.06)**
T stage		
T2 vs T1	**1.96 (*P* = 0.21)**	**0.29 (*P* = 0.15)**
T3 vs T1	**2.50 (*P* = 0.14)**	**0.98 (*P* = 0.98)**
Grade		
G2 vs G1	**4.65 (*P* = 0.18)**	**2.09 (*P* = 0.62)**
G3 vs G1	**7.11 (*P* = 0.10)**	**4.61 (*P* = 0.32)**
Margin status	**0.69 (*P* = 0.41)**	**0.86 (*P* = 0.81)**
Recurrence	**0.98 (*P* = 0.96)**	**1.30 (*P* = 0.66)**
Mortality	**0.43 (*P* = 0.054)**	0.13 (*P* = 0.03)^*^

^a^All models are adjusted for other variables listed

^*^P-value significant at ≤ 0.05

Missing values—neoadjuvant treatment (12), race (9)

**Fig. 1:**
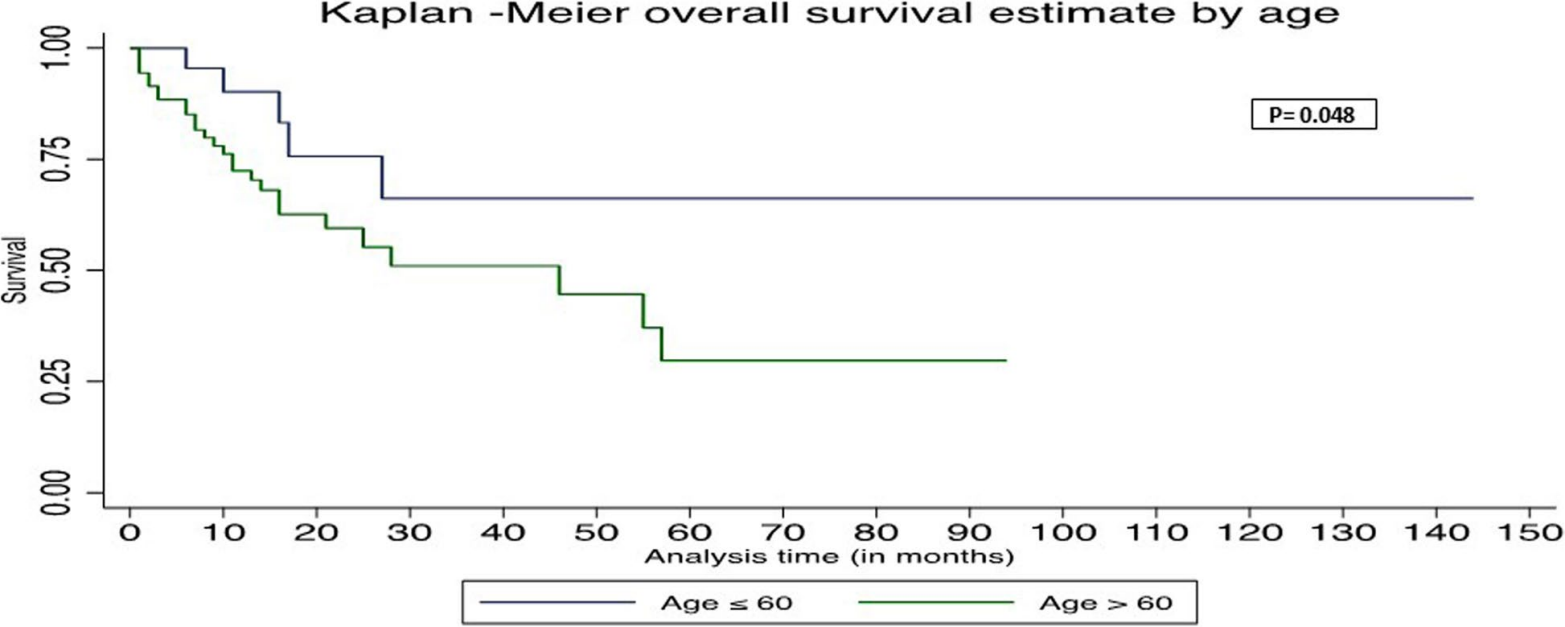
Kaplan–Meier survival graph by overall survival by age

**Fig. 2:**
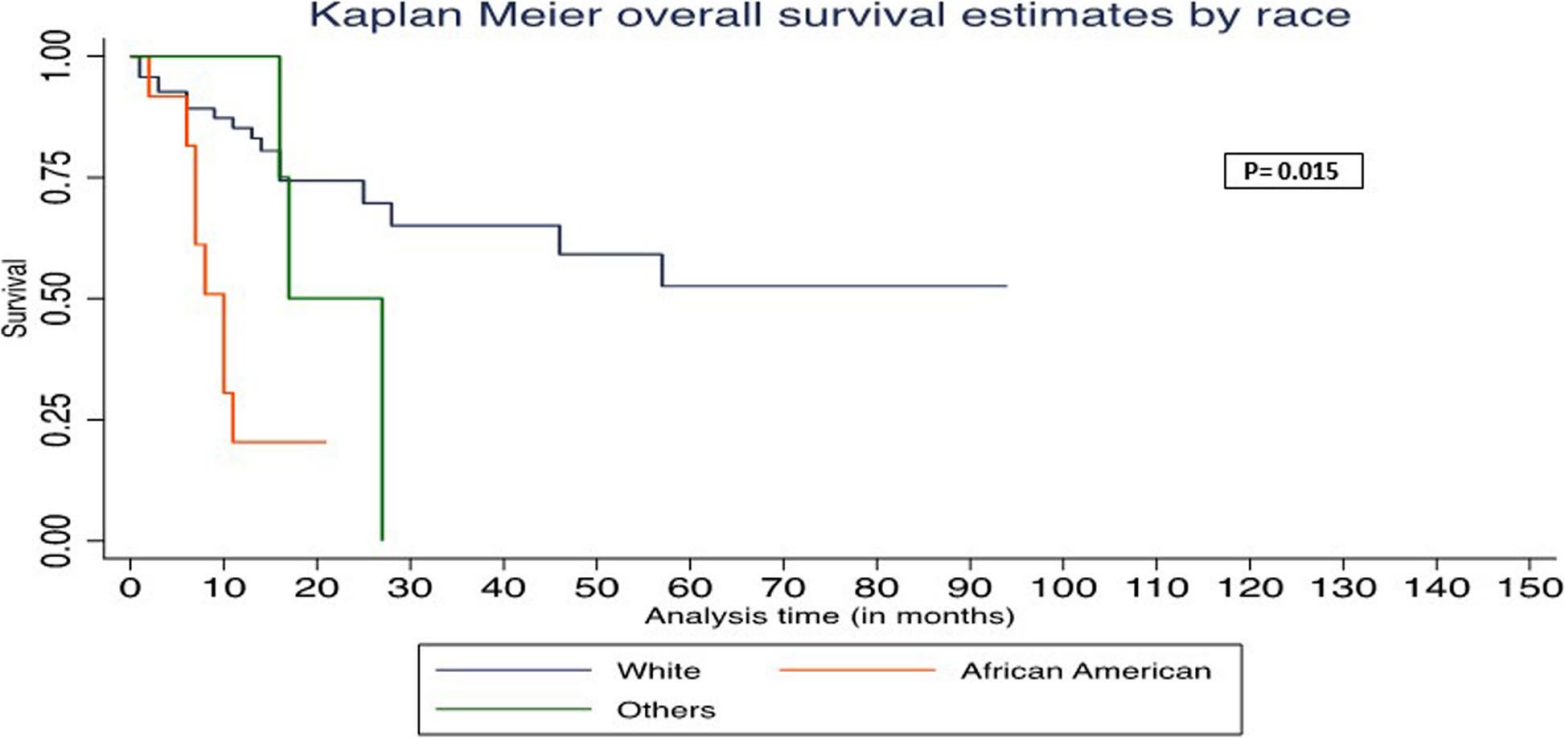
Kaplan–Meier survival graph by overall survival by duodenal invasion

**Fig. 3:**
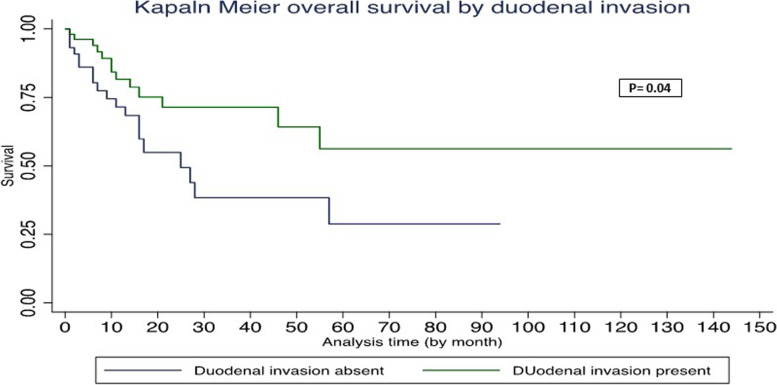
Kaplan–Meier survival graph by overall survival by race

**Fig. 4:**
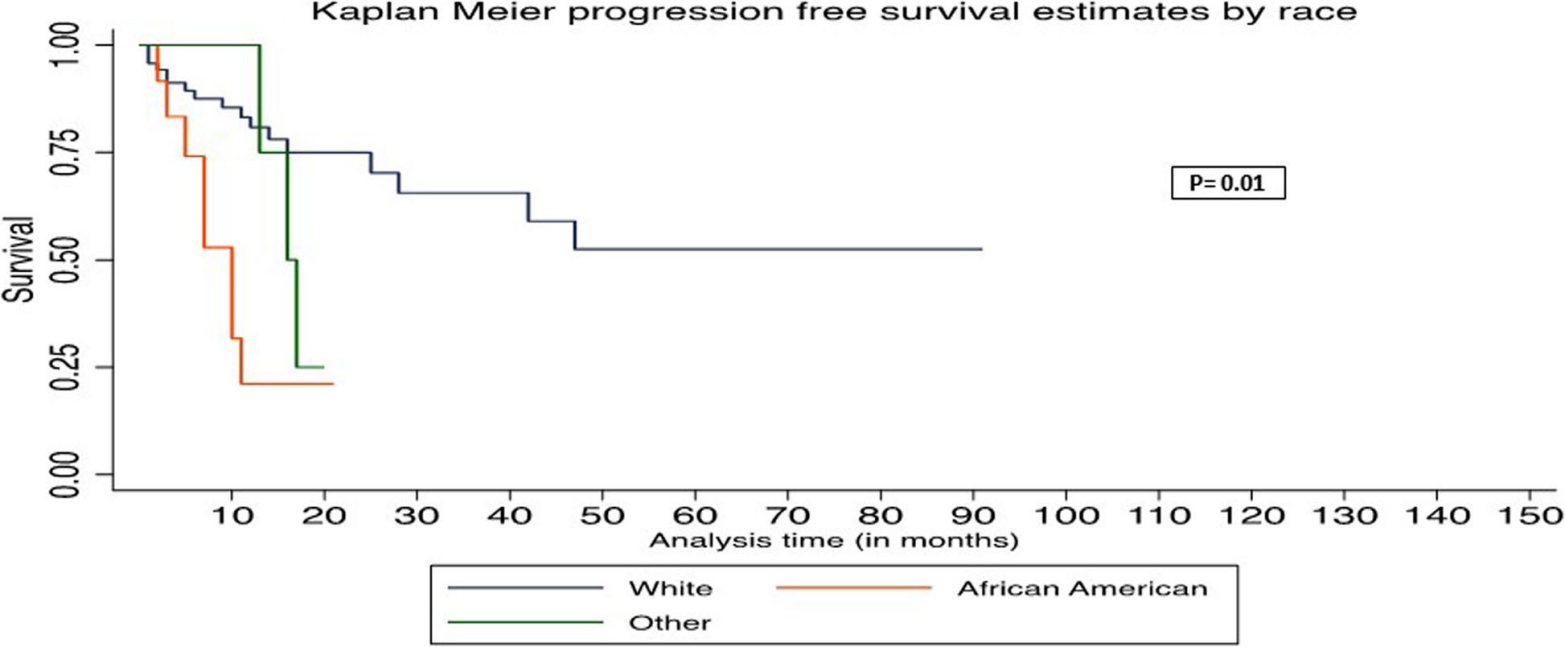
Kaplan–Meier survival graph by progression-free survival by duodenal invasion

**Fig. 5:**
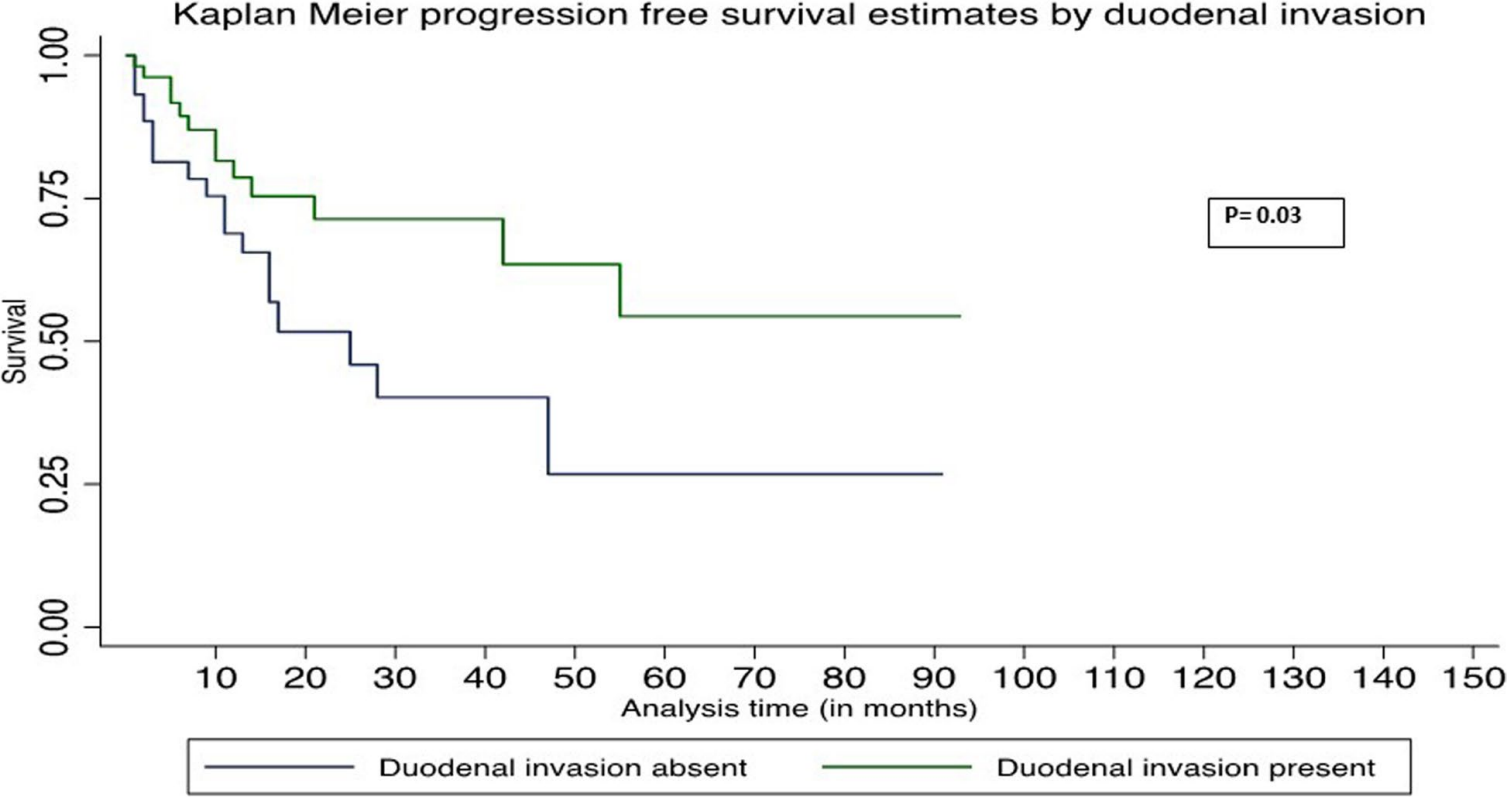
Kaplan–Meier survival graph by progression-free survival by race

In univariate Cox regression analysis, age > 60, absence of duodenal wall invasion, and African American race were associated with worse OS (Table 3). In multivariate Cox regression analysis, age > 60, absence of duodenal wall invasion, and African American race were associated with worse PFS and OS (Table 4). The Kaplan–Meier survival graph by overall survival showed significantly worst survival for age > 60 (*P* = 0.048), African American race (*P* = 0.015), and absence of DWI (*P* = 0.04) (Figs. 1, 2, and 3). The Kaplan–Meier survival graph by progression-free survival was significantly worst for African American race (*P* = 0.01) and absence of DWI (*P* = 0.03) (Figs. 4 and 5). Overall survival for the entire cohort stratified by duration of follow-up and overall progression-free survival for the entire cohort stratified by duration of follow-up are shown in Tables 5 and 6, respectively.

**Table 3:** Univariate Cox regression analysis of adverse factors of pancreatic carcinoma with progression-free survival and overall survival

Characteristics		No. of patients	Progression-free survival		Overall survival	
			HR (95% CI)	*P*	HR (95% CI)	*P*
**Age (y)**	≤ 60 (reference)	26				
	> 60	71	2.39 (0.92–6.22)	0.07	2.63 (1.01–6.87)	0.048*
**Gender**	Female (reference)	39				
	Male	58	0.77 (0.39–1.55)	0.46	0.76 (0.38–1.51)	0.43
**Race**	White (reference)	70				
	African American	12	4.94 (2.02–12.10)	0.00*	5.51 (2.23–13.64)	0.00*
	Other	6	2.27 (0.65–7.98)	0.20	2.14 (0.61–7.51)	0.23
**Tumor stage**	T1 (reference)	20				
	T2	53	0.77 (0.33–1.79)	0.58	0.75 (0.32–1.73)	0.49
	T3	24	1.16 (0.45–2.94)	0.76	1.21 (0.47–3.07)	0.69
**Tumor grading**	G1	5				
	G2	67	0.66 (0.19–2.27)	0.51	0.67 (0.19–2.34)	0.54
	G3	25	1.22 (0.33–4.48)	0.76	1.16 (0.32–4.24)	0.81
**Lymph node involved**	No (reference)	26				
	Yes	71	1.11 (0.51–2.41)	0.78	1.09 (0.50–2.35)	0.84
**Duodenal invasion**	Negative (reference)	44				
	Positive	53	0.47 (0.23–0.95)	0.03*	0.48 (0.23–0.97)	0.04*
**Lymphovascular invasion**	Negative (reference)	27				
	Positive	70	1.07 (0.49–2.32)	0.86	1.06 (0.48–2.28)	0.89
**Perineural invasion**	Negative (reference)	15				
	Positive	82	0.65 (0.26–1.59)	0.34	0.62 (0.25–1.53)	0.30
**Ampullary invasion**	Negative (reference)	63				
	Positive	34	0.96 (0.45–2.03)	0.91	0.91 (0.43–1.93)	0.82
**CBD invasion**	Negative (reference)	79				
	Positive	18	0.94 (0.36–2.45)	0.89	0.85(0.33–2.21)	0.74
**Margin status**	Negative (reference)	68				
	Positive	29	1.69 (0.81–3.57)	0.16	1.59 (0.76–3.33)	0.21

*P-value significant at ≤ 0.05

Missing values—neoadjuvant treatment (12)

**Table 4:** Multivariate Cox regression analysis of adverse factors of pancreatic carcinoma with progression-free survival and overall survival

Characteristics		No. of patients	Progression-free survival		Overall survival	
			HR (95% CI)	*P*	HR (95% CI)	*P*
**Age (y)**	≤ 60 (reference)	26				
	> 60	71	6.37 (1.63–24.81)	0.008*	7.06 (1.76–28.32)	0.006*
**Gender**	Female (reference)	39				
	Male	58	1.34 (0.48–3.73)	0.57	1.29(0.46–3.69)	0.63
**Race**	White (reference)	70				
	African American	12	5.17 (1.62–16.58)	0.006*	5.96 (1.84–19.27)	0.003*
	Other	6	2.92 (0.49–17.41)	0.24	2.59 (0.39–17.00)	0.32
**Tumor stage**	T1 (reference)	20				
	T2	53	0.53 (0.15–1.89)	0.96	0.59 (0.16–2.19)	0.43
	T3	24	0.89 (0.23–3.43)	0.87	1.06 (0.27–4.18)	0.94
**Tumor grading**	G1	5				
	G2	67	0.90(0.14–5.55)	0.91	1.01 (0.16–6.36)	0.99
	G3	25	2.21 (0.32–15.11)	0.42	1.99 (0.28–14.09)	0.49
**Lymph node involved**	No (reference)	26				
	Yes	71	2.05 (0.43–9.78)	0.37	1.89 (0.39–9.20)	0.43
**Duodenal invasion**	Negative (reference)	44				
	Positive	53	0.16 (0.05–0.56)	0.004*	0.19 (0.05–0.64)	0.008*
**Lymphovascular invasion**	Negative (reference)	27				
	Positive	70	1.20 (0.25–5.65)	0.82	0.98 (0.21–4.58)	0.98
**Perineural invasion**	Negative (reference)	15				
	Positive	82	0.48 (0.12–1.95)	0.31	0.55 (0.14–2.21)	0.39
**Ampullary invasion**	Negative (reference)	63				
	Positive	34	3.39 (0.79–14.45)	0.09	2.77 (0.66–11.69)	0.17
**CBD invasion**	Negative (reference)	79				
	Positive	18	0.94 (0.22–4.02)	0.93	0.91 (0.21–3.85)	0.89
**Margin status**	Negative (reference)	68				
	Positive	29	1.45(0.59–3.51)	0.41	1.44 (0.58–3.56)	0.43

*P-value significant at ≤ 0.05

**Table 5:** Overall survival for the entire cohort stratified by duration of follow-up

Variables	≤ 6-month overall survival			6-month–1-year overall survival			1–2-year overall survival			> 2-year overall survival		
	*n*	Mean (95% CI)	*P*-value	*n*	Mean (95% CI)	*P*-value	*n*	Mean (95% CI)	*P*-value	*n*	Mean (95% CI)	*P*-value
**Duodenal invasion**												
** Yes**	13	3.85 (2.7–4.9)	0.15	11	9.45 (8.3–10.6)	0.95	17	18.05 (16.2–19.9)	0.008*	12	69.58 (49.5–89.7)	0.06
** No**	16	2.88 (2.0–3.7)		5	9.4 (6.8–11.9)		13	14.85 (13.7–16.0)		10	45.6 (28.1–63)	
**Age**												
** ≤ 60**	7	4.43 (3.0–5.8)	0.05*	2	9 (3.7–21.7)	0.71	9	16.11 (13.7–18.5)	0.57	8	74.63 (43.7–105.5)	0.06
** > 60**	22	2.95 (2.2–3.7)		14	9.5 (8.4–10.6)		21	16.90 (15.3–18.6)		14	49.57 (36.4–62.7)	
**Lymphovascular invasion**												
** Yes**	21	3.38 (2.5–4.2)	0.74	14	9.8 (8.9–10.7)	0.3	20	16.4 (14.9–18.0)	0.63	15	59.3 (45.0–73.7)	0.89
** No**	8	3.13 (1.8–4.5)		2	7 (7–7)		10	17.1 (14.5–19.7)		7	57.29 (19.4–95.1)	
**Perineural invasion**												
** Yes**	25	3.48 (2.7–4.2)	0.20	13	9.92 (9.0–10.9)	0.01*	24	16.5 (15.2–17.9)	0.69	20	60.7 (46.2–75.2)	0.34
** No**	4	2.25 (0.1–4.6)		3	7.3 (5.9–8.8)		6	17.16 (12.5–21.8)		2	38.5 (7.62–184.6)	
**Lymph node involved**												
** Yes**	22	3.45 (2.6–4.3)	0.45	14	9.71 (8.7–10.7)	0.09	21	17.1 (15.4–18.8)	0.30	14	56.85 (42.2–71.3)	0.72
** No**	7	2.86 (2.0–3.7)		2	7.5 (1.1–13.9)		9	15.66 (13.6–17.8)		8	61.88 (28.4–95.4)	
**Mortality status**												
** Deceased**	11	2.91 (1.5–4.3)	0.35	8	9.13 (7.8–10.5)	0.49	7	16.14 (13.8–18.5)	0.65	6	39.67 (24.2–55.1)	0.07
** Alive**	18	3.56 (2.8–4.3)		8	9.75 (8.2–11.3)		23	16.82 (15.2–18.4)		16	65.81 (48.7–82.9)	

*P-value significant at ≤ 0.05

**Table 6:** Overall progression-free survival for the entire cohort stratified by duration of follow-up

Variables	≤ 6-month progression-free survival			6-month–1-year progression-free survival			1–2-year progression-free survival			> 2-year progression-free survival		
	*n*	Mean (95% CI)	*P*-value	*n*	Mean (95% CI)	*P*-value	*n*	Mean (95% CI)	*P*-value	*n*	Mean (95% CI)	*P*-value
**Duodenal invasion**												
** Yes**	13	3.69 (2.6–4.8)	0.03*	11	7.73 (5.9–9.5)	0.35	17	17 (14.5–19.5)	0.10	12	59.66 (44.7–74.7)	
** No**	16	2.44 (1.9–2.9)		5	9 (7.0–10.9)		13	14.46 (12.9–16.0)		10	39.4 (23.2–55.6)	0.05*
**Age**												
** ≤ 60**	7	3.71 (2.7–4.7)	0.16	2	9 (3.7–21.7)	0.60	9	15 (11.7–18.3)	0.45	8	54.1 (28.9–79.3)	
** > 60**	22	2.77 (2.1–3.5)		14	8 (6.5–9.5)		21	16.28 (14.4–18.2)		14	48.4 (35.5–61.2)	0.62
**Lymphovascular invasion**												
** Yes**	21	3.09 (2.3–3.9)	0.59	14	8.28 (6.8–9.8)	0.5	20	15.45 (13.4–17.5)	0.42	15	55.6 (40.4–70.8)	0.16
** No**	8	2.75 (1.8–3.7)		2	7 (7–7)		10	16.8 (14.1–19.5)		7	39.42 (26.3–52.5)	
**Perineural invasion**												
** Yes**	25	3.12 (2.5–3.8)	0.30	13	8.31 (6.7–9.9)	0.54	24	15.6 (13.9–17.4)	0.55	20	52.35 (40.8–63.9)	0.27
** No**	4	2.25 (0.1–4.6)		3	7.3 (5.9–8.8)		6	16.83 (11.8–21.9)		2	31.5 (17.5–202.56)	
**Lymph node involved**												
** Yes**	22	3.04 (2.3–3.8)	0.78	14	8.21 (6.7–9.7)	0.71	21	16.09 (13.9–18.2)	0.70	14	53.07 (37.7–68.4)	0.52
** No**	7	2.86 (2.0–3.7)		2	7.5 (1.1–13.9)		9	15.4 (13.10–17.8)		8	45.87 (27.1–64.6)	
**Mortality status**												
** Deceased**	11	2.27 (1.3–3.3)	0.04*	8	8 (6.1–9.9)	0.84	7	15.29 (12.2–18.4)	0.66	6	35 (18.6–51.4)	0.07
** Alive**	18	3.4 (2.7–4.2)		8	8.25 (6.0–10.5)		23	16.08 (14.2–18.0)		16	56.3 (42.6–69.9)	

*P-value significant at ≤ 0.05

Of our cases, 15 patients (15%) had IPMN associated with adenocarcinoma. Of patients with invasive IPMN, 47% had DWI, while 56% of patients without IPMN had DWI. The presence of extrapancreatic bile duct involvement was not predictive of survival or recurrence (Table 4).

## Discussion

In our cohort, 55% of pancreatic head carcinoma patients had DWI, and 19% had extrapancreatic bile duct involvement. The presence of DWI was not associated with inferior recurrence-free or overall survival. In contrast, our cohort showed a better survival outcome even on multivariate analysis. Possible explanations include early clinical presentation of patients with DWI such as jaundice, nausea, and early satiety from delayed gastric emptying due to gastric outlet obstruction; inclusion of patients who received chemotherapy treatment; and differences in clinicopathologic and demographic characteristics of the patient population under study. Also, we had a smaller sample size and the validity of this data needs to be further substantiated in larger cohort studies. A preoperative imaging-based study found that DWI was associated with lower survival after Whipple surgery [9]. However, the study lacks correlation with expert pathology review for duodenal invasion and other adverse factors such as lymph node involvement on resection. Similar to prior reports, our study also showed that African American patients had worse PFS and OS, which might be related to patients’ demographics, treatment received, and access to health care [10, 11]. In a series of 223 consecutive pancreaticoduodenectomies for pancreatic adenocarcinoma, 74% of cases showed duodenal involvement by pathologic evaluation, but it did not have any association with clinical outcome [8]. However, Dal Molin et al. reported 45.2% duodenal involvement in a cohort of 1128 pancreatic carcinoma patients who did not receive neoadjuvant therapy, and this was found to be an independent negative prognostic factor with inferior survival [7]. However, their cohort included pancreaticoduodenectomies with or without total pancreatectomy as well as distal pancreatectomy specimens, and DWI was only detected in patients with pancreatic head carcinoma. Distal pancreatectomies, by nature of the specimen, will not have a segment of the duodenum to assess for DWI, and these patients have different prognostic parameters from pancreatic head adenocarcinoma. For example, in a multi-institutional study of 454 distal pancreatic cancer patients, different adverse factors were found such as the presence of non-IPMN invasive carcinoma, splenic artery invasion, venous invasion, splenic parenchymal invasion, pT3 stage (AJCC 8th edition), and lymph node involvement [12]. This may have contributed to the observed adverse outcome associated with duodenal wall invasion in their study. To avoid this dissimilarity in our patient cohort, we excluded 23 patients with adenocarcinoma who underwent distal pancreatomies during this time period. Analysis of pancreatic head adenocarcinoma and distal pancreatectomy patients in our cohort did not show any impact of duodenal wall invasion on PFS nor OS (in univariate as well as multivariate analysis, data not shown). The variation in the existing studies and the disparities in the results highlight the need for validating the usage of a single staging system for both pancreatic head and distal pancreas adenocarcinoma patients.

The 7th edition of the AJCC had been criticized for its definition of pT3 pancreatic adenocarcinoma and for including all lymph node metastasis as pN1. In the 7th edition, the presence of extrapancreatic invasion, including duodenal wall invasion, was staged as pT3 regardless of tumor size. However, there is inconsistency among pathologists in assessing tumoral extension beyond the pancreas, as there are no well-defined boundaries to the pancreas. In addition, Saka et al. found that 91% of pancreatic carcinomas invade into peripancreatic tissue and would be staged as pT3 in the old system by the “orange peel” grossing technique examining the entire soft tissue covering the pancreas [8]. Comparing the 7th edition AJCC with the 8th edition is beyond the scope of the study given the interobserver variability in assessing extrapancreatic soft tissue invasion. In addition, we did not use the “orange peel” technique and only representative sections were submitted in the majority of cases (1 section per 1 cm of lesion), so there is a possibility of under-staging cases according to the 7th edition AJCC.

We did not find the 8th edition AJCC tumor staging to be predictive of outcome in our cohort. However, it was shown to correlate with survival in a large cohort of treatment-naive patients from three large US pancreatic centers (2318 R0 pancreatic carcinoma patients) [13]. Although the 8th edition AJCC staging is more reproducible, some studies did not find it better than the 7th edition AJCC in stratifying pancreatic carcinoma patients [12, 14]. For example, Fan et al. found the 8th edition AJCC staging not predictive of survival in patients with invasive IPMN with tumor size > 2 cm (i.e., comparing pT2 versus pT3) [14]. In a series of 454 patients with resected distal pancreatic adenocarcinoma, there was no significant difference in PFS or OS between pN1 and pN2, but both 7th and 8th edition pT stages were predictive of survival [12].

Our cohort included 27 patients who received neoadjuvant chemotherapy with or without radiotherapy, but none of them showed a pathologic complete response (i.e., no residual tumor). Inclusion of this subset of patients in our study may have impacted survival analysis, as tumor regression and therapy-related changes may prevent accurate assessment of the tumor size during gross or microscopic examination, and larger histologic sections would facilitate the assessment of treatment response [15, 16]. In addition, survival is longer with an extended duration of chemotherapy and major pathologic response [16, 17]. Although the 8th edition AJCC staging was found to be applicable in the resected patient following neoadjuvant therapy, Chatterjee et al. found no difference in stratifying patients with tumor size > 1 cm (i.e., no difference between ypT1c, ypT2, or ypT3) in a series of 398 resected patients and suggested using 1 cm as a cutoff for ypT2, and the majority of ypT3 cases (according to the 7th AJCC edition) were down-staged to either ypT1 or ypT2 using the tumor size in the 8th AJCC [18].

Regarding lymph node stage, the previous AJCC edition defined any lymph node involvement as pN1 regardless of number, while the 8th edition added a pN2 category for patients with metastasis in more than three lymph nodes, which was shown to be prognostically significant in some studies [11]. However, a prognostic significance of pN staging in the 8th edition based on the number of involved lymph nodes was not demonstrated in some studies [12, 19].

Limitations of our study are the retrospective analysis from a single institution experience. Multicenter collaboration to evaluate the significance of duodenal invasion in a larger cohort of pancreatic head adenocarcinoma patients would be necessary to confirm our observation and evaluate the impact on survival and recurrence.

In conclusion, our study showed that duodenal wall invasion is associated with increased lymph node metastasis. However, it is not associated with inferior overall survival, justifying the AJCC 8th edition’s decision to exclude this parameter from the pancreatic carcinoma tumor staging protocol.

## Data Availability

The data is accessed and stored at a secure university cancer database.
